# Probabilistic models for CRISPR spacer content evolution

**DOI:** 10.1186/1471-2148-13-54

**Published:** 2013-02-26

**Authors:** Anne Kupczok, Jonathan P Bollback

**Affiliations:** 1 IST Austria (Institute of Science and Technology Austria), Am Campus 1, A-3400 Klosterneuburg, Austria

**Keywords:** CRISPR/Cas, Maximum Likelihood, Microbial genome evolution, Bacterial immunity

## Abstract

**Background:**

The CRISPR/Cas system is known to act as an adaptive and heritable immune system in Eubacteria and Archaea. Immunity is encoded in an array of spacer sequences. Each spacer can provide specific immunity to invasive elements that carry the same or a similar sequence. Even in closely related strains, spacer content is very dynamic and evolves quickly. Standard models of nucleotide evolution cannot be applied to quantify its rate of change since processes other than single nucleotide changes determine its evolution.

**Methods:**

We present probabilistic models that are specific for spacer content evolution. They account for the different processes of insertion and deletion. Insertions can be constrained to occur on one end only or are allowed to occur throughout the array. One deletion event can affect one spacer or a whole fragment of adjacent spacers. Parameters of the underlying models are estimated for a pair of arrays by maximum likelihood using explicit ancestor enumeration.

**Results:**

Simulations show that parameters are well estimated on average under the models presented here. There is a bias in the rate estimation when including fragment deletions. The models also estimate times between pairs of strains. But with increasing time, spacer overlap goes to zero, and thus there is an upper bound on the distance that can be estimated. Spacer content similarities are displayed in a distance based phylogeny using the estimated times.

We use the presented models to analyze different *Yersinia pestis* data sets and find that the results among them are largely congruent. The models also capture the variation in diversity of spacers among the data sets. A comparison of spacer-based phylogenies and Cas gene phylogenies shows that they resolve very different time scales for this data set.

**Conclusions:**

The simulations and data analyses show that the presented models are useful for quantifying spacer content evolution and for displaying spacer content similarities of closely related strains in a phylogeny. This allows for comparisons of different CRISPR arrays or for comparisons between CRISPR arrays and nucleotide substitution rates.

## Background

Bacteria and Archaea have an adaptive heritable immune system against viruses, plasmids and other mobile genetic elements [1,2]. This locus, CRISPR (Clustered Regularly Interspaced Short Palindromic Repeats), consists of an array of repeats and unique spacers. The repeats are of length 21-48 nucleotides depending on CRISPR type and species. The spacer sequences are 26-72 nucleotides in length, where the variance of spacer length within one array is small. The spacer sequences were found to be of extrachromosomal origin [3] and are involved in immunity [1,2]. Cas (CRISPR-associated) genes adjacent to the CRISPR arrays are necessary for the biogenesis of the CRISPR RNA, for the interference with the target nucleic acid and for the acquisition of new spacer sequences [4]. Different types of CRISPR/Cas systems exist based on the set of Cas genes present [5].

Comparisons of the CRISPR array of closely related strains showed that the CRISPR array undergoes a rapid evolution that is mainly determined by the gain and loss of the whole system or of individual spacers [6,7]. In most cases, spacer addition was observed at the beginning, the ‘leader’ end, of the array [1] and the pattern in metagenomic samples suggest that deletion of consecutive repeat-spacer units occurs [6]. Bacterial genomes can have multiple CRISPR arrays that differ in their dynamics [7-9]. It was observed that closely related strains can differ in their spacer content, thus the CRISPR array is used as a tool for strain typing (e.g., [8,10,11]).

The targeting of extrachromosomal elements by the CRISPR/Cas system was discovered recently [1,2] and many questions regarding the functions, mechanisms and evolution of this locus are still open. This is complicated by the fact that different CRISPR/Cas systems have different mechanisms and may have different function [4]. Thus computational methods that make predictions are important to narrow the space of hypotheses that need to be tested experimentally. For example, self-targeting spacers are not conserved between species and CRISPR arrays with self-targeting spacers may get inactivated. These observations exclude the hypothesis of gene regulation by CRISPR [12].

Using model simulations can provide insights into the parameters allowing CRISPR existence and into the details of CRISPR dynamics. One result using population genetics models is that CRISPR is maintained if it provides immunity to viruses or plasmids even when there is a cost of having CRISPR [13]. Simulating a spatial model of virus and host population showed that coexistence is possible with a CRISPR-based immune system [14]. Furthermore, a spatially structured environment can lead to intermediate array lengths, i.e., the number of spacers has an optimum between 0 and the number of viruses excluding the extreme values. Then the lengths are determined by the spacer insertion rate and by the cost for having spacers not by the total number of phages in the environment [15]. Modeling coevolution of hosts and viruses results in the observation that spacers at the leader-distal end tend to be more conserved, due to selective sweeps, and that immunity to contemporary viruses is mainly determined by the most recently acquired spacers [16,17]. In addition, simulations can find parameter regimes that are important for the existence of CRISPR like a threshold on the viral mutation rate [18].

Our approach differs from the population genetics models described since it *estimates* parameters directly from the array data. We describe the dynamics of the CRISPR locus over time in diverging populations related by a phylogeny. This is the phylogeny of the CRISPR/Cas locus. Since the locus can be transferred horizontally [19], the CRISPR/Cas phylogeny does not need to be identical to the strain phylogeny. There are a few instances of recombination inside cas genes [20], but in our model, we exclude recombination in the spacer arrays. The evolutionary events we model are spacer insertion and deletion. By using only strains harboring the locus, we ignore the loss or gain of the whole CRISPR/Cas system.

 Mutations inside the CRISPR locus are also not included in the model, but in data analyses multiple spacers with sequence similarities can be subsumed into one identity.

Even before the function of the CRISPR/Cas system was clear, Pourcel et al. [8] formulated three observations for CRISPR evolution by comparing *Yersinia pestis* arrays: Random deletions of one or more spacers and repeats; polarized addition of new spacers; and identical spacers reflect shared ancestry not independent events. We also assume that the CRISPR arrays analyzed are homologous and that each spacer was only inserted once, i.e., all spacers with identical sequence are identical by descent. Thus we present three models: an unordered model (spacer content is considered as a set), an ordered model (where insertion is polarized, i.e., insertions occur at one end only) and a fragment loss model (where insertion is polarized and successive spacers can get deleted together in a single event).

Another class of models that take order relationships into account are gene order models, i.e., they model the order of genes in the genome over time. Most methods for evaluating the distance between two gene orders find the minimum number of rearrangement events between these genomes. This approach can also be combined with insertions and segment deletions [21,22]. Probabilistic methods of rearrangement only model inversions [23,24] or inversions and transpositions [25]. Multi-gene events are considered in one model of gene innovation, duplication and deletion, but ignoring the order of genes on the genome [26].

Our ordered and fragment loss models are thus different from the probabilistic models for gene order since they capture the properties specific for CRISPR spacer evolution. We describe our method and investigate its properties by simulation and application to real world *Yersinia pestis* data sets [8,27].

## Methods

### Models

**Figure 1:**
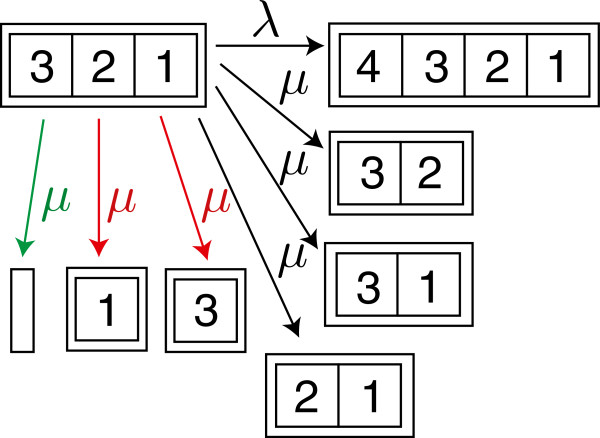
**Illustration of the instantaneous rates for an array of length 3.** Leader-proximal end is on the left. The arrows display the allowed transitions for the fragment loss model. Deletions of length two are are displayed in red, deletions of length three in green. For the independent loss model, only the black arrows are allowed transitions. For the unordered model, the transition with rate *λ* results in either 4-3-2-1, 3-4-2-1, 3-2-4-1 or 3-2-1-4 with uniform probabilities.

We describe different models for estimating insertion and deletion rates from CRISPR arrays. We ignore repeats and only use the spacer information and their order encoded in an array. The leader end is displayed on the left (see also Figure 1). In our models, these arrays evolve by insertion and deletion events. An overview of the types of insertions and deletions allowed in the different models can be found in Table 1. In all models, the waiting time for insertion events is exponentially distributed with rate *λ* (Figure 1). One spacer is inserted for each insertion event.

In the *independent loss model* only single spacers can get lost. For each spacer, the waiting time to get lost is exponentially distributed with rate

**Table 1:** Overview over models

	**Independent loss model**		**Fragment loss model**
	**Unordered**	**Ordered**	
Insertions	Random	Polarized	Polarized
Deletions	Single	Single	Fragments

**Figure 2:**
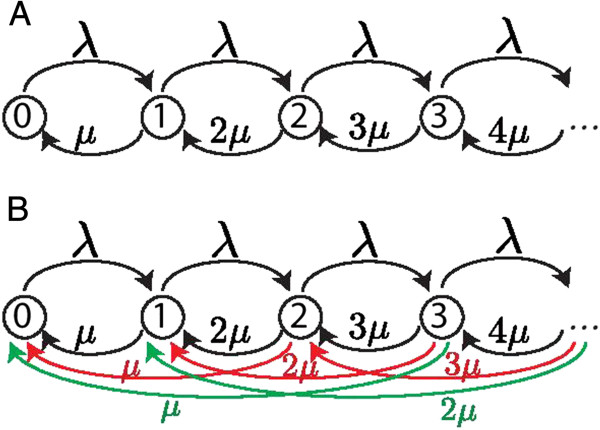
**Markov chain representation of the length models.** (**A**) Independent loss model. (**B**) Fragment loss model. For clarity, deletions of length 2 are red, deletions of length 3 are green, and deletions ≥4 are not displayed.

*μ*. All deletions are independent of each other. The corresponding *length model* describes the length of the array by a Markov process (Figure 2). In contrast, the *full model* takes spacer identities into account. In the independent loss model, a loss means a transition to length -1 and a gain a transition to length +1. We analyze two sub-models of the independent loss model: the *unordered* model, where there is no position information; and the *ordered model*, where insertion occurs in a polarized way, i.e., at the end adjacent to the leader. For simplicity, we refer to this end as the *beginning* of the array. The latter model is motivated by the observation that spacers are usually inserted at the leader end of the array (e.g., [1]).

In the *fragment loss model* the position is informative since insertion occurs in the beginning and subsequent spacers can get lost together. This model is motivated by the pattern in metagenomic samples that shows deletion of consecutive repeat-spacer units [6]. Each possible non-empty substring of the array is a fragment. Thus fragments can be overlapping and one spacer inside an array is then part of different fragments. For example, the array 3-2-1 (Figure 1) consists of the fragments 1, 2, 3, 2-1, 3-2 and 3-2-1. The fragment 3-2 overlaps with the fragment 2-1 in the spacer 2. And the spacer 2 occurs in 4 fragments: 2, 2-1, 3-2 and 3-2-1. For each possible fragment, the waiting time to get lost is exponentially distributed with rate *μ*, independent of the number of spacers a fragment contains. In the length model, all lengths smaller than the current length are accessible in a single step (Figure 2).

Since *μ* has a different meaning in both models, we emphasize this by using *μ*_*F*_ for the fragment loss model (*μ*_*F*_ affects each possible fragment), and *μ*_*I*_ for the independent loss model (*μ*_*I*_ affects only single spacers). The rates are always rescaled such that one event (insertion or deletion) is expected in time *t* = 1. This allows for estimating times, but only the ratio $\rho =\frac{\lambda }{\mu }$ can be estimated. Again, we distinguish the two models by using $\rho_{F}=\frac{\lambda }{\mu_{F}}$ and $\rho_{I}=\frac{\lambda }{\mu_{I}}$. Subscripts are omitted when the underlying model is clearly stated.

Now, we present the stationary distribution of the length models and the transition probabilities of the full model necessary to formulate an estimation approach under each of these models. Afterwards details of the estimation approaches are described.

#### Independent loss models

##### Length model

The independent loss length model is a Markov process known as an *M* / *M* / *∞* queuing model [28] (Figure 2A). In this queuing model, customers (i.e., spacers) arrive according to a Poisson process with rate *λ*. They are immediately served and exit after an exponential waiting time with rate *μ*. The stationary distribution of the number of busy servers (i.e., the number of spacers in the array), is a Poisson distribution with rate *ρ*:

(1) $$p(n|\rho )=e^{-\rho }\frac{\rho^{n}}{n!},\;\text{where}\;n\;\text{is the array length}.$$

##### Transition probabilities

Given an ancestor *s*_0_ and a descendent *s*_1_, *m* spacers are shared, *d* spacers are unique to *s*_0_ and *j* spacers are unique to *s*_1_. The transition probabilities of changing from *s*_0_ to *s*_1_ use the property that inserted, preserved and deleted elements are independent of each other:

(2) $$\;\begin{array}{c} T(s_{0}\to s_{1}|t,\lambda ,\mu )=M(m|t,\mu )D(d|t,\mu )I(j|t,\lambda ,\mu )\\ \text{Where the probability of preserving}\;m\;\text{spacers in time}\\ \;t\;\text{is}\;M(m|t,\mu )=e^{-m\mu t},\\ \text{the probability of loosing}\;d\;\text{spacers in time}\;t\;\text{is}\\ \;D(d|t,\mu )={\left(1-e^{-\mu t}\right)}^{d},\\ \text{the probability of inserting}\;j\;\text{spacers in time}\;t\;\text{is}\;\\ \;I(j|t,\lambda ,\mu )=\frac{e^{-\rho \left(1-e^{-\mu t}\right)}{\left(\rho \left(1-e^{-\mu t}\right)\right)}^{j}}{j!}. \end{array}$$

*M* and *D* follow directly from the exponential model. *I* is known from queuing theory [28]. The probability of inserting *j* spacers is the probability of observing *j* spacers after time *t* when there were 0 spacers at time 0. That is the integration over all possible paths leading to *j*, including paths where spacers were inserted and lost and thus never observed.

#### Fragment loss models

##### Length model

The stationary distribution of the length model (Figure 2B) is given by

(3) $$p(n|\rho )=\frac{\left(n+1)(n+2\right)}{2\rho \overset{n}{\underset{i=0}{\prod }}(\frac{\left(i+1)(i+2\right)}{2\rho }+1)}.$$

Equation (3) can be solved from the conditions that in stationarity the flow into a state equals the flow out of that state and that the probabilities of such events necessarily sum to 1 (see Additional file 1).

**Figure 3:**
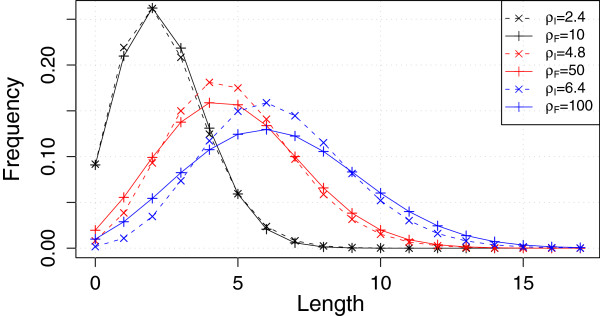
**Stationary distribution of the length models.** Subscript I represents the independent loss model and subscript F the fragment loss model. *ρ*s of the same color result in the same mean length, i.e., they are corresponding *ρ*s.

For each *ρ*_*F*_ there is a *correspondingρ*_*I*_ that has the same expected length. We find that for corresponding *ρ*s the fragment loss model has a higher variance of the length distribution than the independent loss model (Figure 3).

##### Transition probabilities

**Figure 4:**
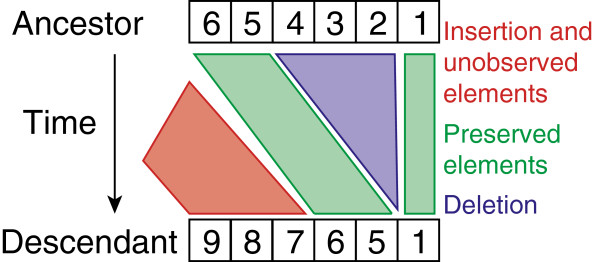
**Overview of the array segmentation for the likelihood calculation under the fragment loss model.** This segmentation results in the inserted fragment 9-8-7, the preserved fragments 6-5 and 1 and the deleted fragment 4-3-2.

Given an ancestor *s*_0_ and a descendent *s*_1_, we segment them into independent pairs (Figure 4). Note that this segmentation is different from the fragments described above. Fragments are all possible substrings of one array, but segments are calculated using two arrays. Each segment is either an inserted, deleted or preserved segment. Segments are of maximal length, i.e., two consecutive segments are of different type. See Figure 4 for an example of segments resulting from a pair of arrays. In contrast to the independent loss model, this segmentation is an approximation since it ignores the probability of deletion events spanning multiple segments. The segmentation is, however, necessary to factorize the transition probabilities. The transition probability is then the product over the segment probabilities.

Preserving a fragment of length *m* has probability

$$\begin{array}{c} M(m|t,\mu )=e^{-\frac{m(m+1)}{2}\mu t}. \end{array}$$

Deleting a fragment of any length has probability

$$\begin{array}{c} D(t,\mu )=1-e^{-\mu t}. \end{array}$$

Inserting *i* spacers has probability

(4) $$\begin{array}{c} \;I(i|t,\lambda ,\mu )=2^{i+1}e^{-\mathrm{\lambda t}}\rho^{i}\\ \;\;\;\;\times \overset{i+1}{\underset{k=1}{\sum }}\left[{\left(-1\right)}^{k-1}\frac{\left(1+2k)k(k+1\right)}{2(i-k+1)!(i+k+2)!}\right)\\ \;\;\;\;\times \left(\frac{e^{-\frac{k(k+1)}{2}\mu t}\left(1-\frac{(i+2)(i+1)-(k+1)k}{2\rho }\right)}{1+\frac{k(k+1)}{2\rho }})\right]\\ \;\;\;\;+\frac{\left(i+1)(i+2\right)}{2\rho \overset{i}{\underset{k=0}{\prod }}\left[\frac{\left(k+1)(k+2\right)}{2\rho }+1\right]}. \end{array}$$

As before, the probability of inserting *i* spacers includes unobserved spacers that were inserted and lost again. These equations were found by integration over all possible paths in Mathematica 8.0 [29].

For example, in Figure 4 the transition probability is *I*(3|*t*, *λ*, *μ*) × *M*(2|*t*, *μ*) × *D*(*t*, *μ*) × *M*(1|*t*, *μ*).

### Estimation

#### Maximum likelihood function

We describe a maximum likelihood approach to estimate rates and times of spacer insertions and deletions, given a set of ordered spacer arrays from different strains. Since we do not have phylogenetic information, we consider each pair of arrays and their possible common ancestors.

Formally, the maximum likelihood estimate for a spacer set *S* with k = |*S*| is

(5) (ρ^,t^)=argmaxL(ρ,t|S)withL(ρ,t|S)=∏i=1,…,k2L(ρ,ti|si),

where ***s*** is the list of all different pairs of *S* and ***t*** is the corresponding list of pairs of times.

The likelihood of a pair of spacer arrays (*s*_1_, *s*_2_) with times (*t*_1_, *t*_2_) is then

(6) $$\;\begin{array}{c} L(\rho ,t_{1},t_{2}|s_{1},s_{2})=\underset{\text{ancestors}\;a}{\sum }q(a|\lambda ,\mu )T(a\to s_{1}|t_{1},\lambda ,\mu )\\ \;\times T(a\to s_{2}|t_{2},\lambda ,\mu ), \end{array}$$

where *λ* and *μ* are computed from *ρ* given the constraints $\frac{\lambda }{\mu }=\rho$ and the expected number of insertions and deletions in time 1 is 1. Then, *q*(*a*|*λ*, *μ*) is the probability of observing *a*, *T*(*a* → *b*|*t*, *λ*, *μ*) is the transition probability of changing from *a* to *b* in time *t* given insertion rate *λ* and deletion rate *μ*.

If the pair has no overlap, i.e., no common spacers, we assume that the time from the common ancestor is long enough such that the transition probabilities approach the stationary probabilities. Then the likelihood function can be simplified by using the fact that the probability of the whole ancestor space is 1. We find that only the lengths are informative for estimating *ρ*:

(7) $$\;\begin{array}{c} L(\lambda ,\mu |s_{1},s_{2})=\underset{\text{ancestors}\;a}{\sum }q(a|\lambda ,\mu )p(n_{1}|\rho )p(n_{2}|\rho )\\ \;=\;p(n_{1}|\rho )p(n_{2}|\rho ),\text{with}\;n_{1}\;=\;|s_{1}|\text{and}\;n_{2}\;=\;|s_{2}|. \end{array}$$

Note that *q* and *p* are different but related by the following constraints: The sum of all *q*(*a*) with |*a*| = *n* is *p*(*n*) and *q*(*a*) = *q*(*b*) if |*a*| = |*b*|.

#### Optimization

We are interested in both the estimate of *ρ*, $\hat{\rho }$, and the estimation of the divergence times. For a pair, we denote the estimated time between two arrays as $\hat{\tau }={\hat{t}}_{1}+{\hat{t}}_{2}$. *τ* for a phylogeny or for a collection of pairs denotes the average of *τ* over all pairs.

Overview of the estimation approach: 

1. Estimate a starting *ρ* from the length model by maximum likelihood. The likelihood function is $L_{\text{start}}(\rho )=\underset{\text{arrays}s}{\sum }p(|s|\;|\rho )$, where |*S*| is the length of *s* and *p* is the stationary distribution of the length model.

2. For each pair of spacers with overlap, generate the possible ancestors: Ancestral arrays can be arbitrarily large, but the probability of observing a certain length is given by *p*(*n*). For practical reasons we do not consider ancestors whose length is outside the central 99% of the stationary distribution given by *ρ* estimated in step 1, since they would have a negligible contribution to the likelihood. In detail, the length *l*_1_ where the cumulative distribution exceeds 0.005 is the minimum ancestor length and the length *l*_2_ where the cumulative distribution exceeds 0.995 is the maximum ancestor length. Then the possible ancestor lengths *n* are between *l*_1_ and *l*_2_: *l*_1_ ≤ *n* ≤ *l*_2_.

3. 

(a) For all pairs with overlap, estimate the times with fixed *ρ*. It is possible to iterate through the pairs and estimate their times independently of the other pairs. The estimation of both times is iterated alternatingly until the likelihood has converged.

(b) Estimate *ρ* with fixed times using *L*(*ρ*|***t***, *S*).

(c) Check if the log-likelihood of the estimated parameters has converged, then return the estimated parameters, else repeat step (a) with the new parameters.

All three models are analyzed in this computational framework. All optimization steps only optimize one parameter and use Powell’s method from the python package scipy[30]. The python package mpmath is used for high-precision computing [31] that is necessary to compute the probability functions accurately.

#### Ancestors

Here, we describe for each of the models how we generate the ancestors in step 2 above. Thereby we must account for unobserved spacers, that are not present in the data but in ancestral lineages. We overcome the problem of the infinite state space by ignoring the identity of unobserved spacers. For example, there may be four unobserved spacers, each of them gets a new unique name, but then no other four unobserved spacers with other names or an other order are considered.

##### Unordered model

Given a pair of arrays *s*_1_ and *s*_2_, they have *c* spacers in common, *d*_1_ are unique to *s*_1_ and *d*_2_ are unique to *s*_2_. Then all *n* between min(*c*, *l*_1_) and *l*_2_ are generated. When length *n* is generated, enumerate all *i*, *j*, *u* such that *c* + *i* + *j* + *u* = *n*, *i* ≤ *d*_1_ and *j* ≤ *d*_2_. Then for ancestor *a*, there are *c* common spacers, *i* only occur in *s*_1_, *j* only occur in *s*_2_ and *u* are unobserved (they are lost in both lineages). Since this ancestor comprises multiple spacer identities, we assign a weight to it, w(a)=d1i×d2j. The weights for each *n* are rescaled such that they sum to 1, i.e., the rescaled weight *w*_*s*_ is $w_{s}(a)=\frac{w(a)}{\underset{b,|b|=|a|}{\sum }w(b)}$. Then *q*(*a*) = *w*_*s*_(*a*)*p*(|*a*|).

##### Ordered model

Given a pair of arrays *s*_1_ and *s*_2_, find the first shared spacer. The ancestor must contain this spacer and all subsequent spacers from both arrays, these are *c* spacers in total. There are *d*_1_ and *d*_2_ spacers before the first shared spacer in *s*_1_ and *s*_2_, respectively. With these new definitions of *c*, *d*_1_ and *d*_2_, the method from the unordered model is applied.

##### Fragment loss model

For the fragment loss model, the ancestors must fulfil several constraints given by the order in the observed arrays. Since all shared ancestors are identical by descent and insertions occurs only in the beginning, all spacers from the first shared spacer on must be present in the ancestor. Thereby the order of spacers must be preserved. Enumerating the ancestors is best explained with an example. Consider the arrays *s*_1_ = 8-7-6-4-3-2-1, *s*_2_ = 11-10-9-7-6-5-2. 

• 7 is the first shared spacer.

• The set of spacers necessarily present in the ancestor is the union of all spacers after the first shared spacer: {1,2,3,4,5,6,7}. 

– Possible orders of these spacers: 7-6-5-4-3-2-1, 7-6-4-5-3-2-1, or 7-6-4-3-5-2-1

• The set of spacers possibly present in the ancestor is the union of all spacers before the first shared spacer: {8,9,10,11}. 

– Order constraints for these spacers: 11 before 10 before 9 before 7

• Unobserved spacers (spacers present in the ancestor and lost in both lineages) may have occurred at all possible positions.

Since a lot of possible arrays are generated by this approach, heuristics are used to reduce their number: 

• Shared fragments cannot be interrupted by an unobserved spacer. 

In the example, there is no unobserved spacer between 6 and 7.

• Unique fragments in the beginning are not mixed. 

– In the example, 8 and 11-10-9 are in the beginning and then the following ancestral fragments are not allowed: 11-8-10 and 10-8-9.

• Deleted pairs are also not mixed. 

– In the example 4-3 and 5 are deleted and the ancestral fragment 4-5-3 is ignored.

• The number of positions with unobserved spacers is maximal four. That means there can still be a lot of unobserved spacers but they occur only in maximal four stretches.

This reduction is only for computational reasons, and may result in the true/simulated ancestor not being included in the set of possible ancestors. For small simulations it was shown that the results are very similar (data not shown) and that the ancestors generated contain enough information for the likelihood function.

#### Loss time

Two arrays do not contain information about the divergence time if they have no overlap. To include them in the analysis, we are interested in the time passed until an array lost all spacers present in the ancestor.

The *lineage loss time distribution* for a given *ρ* is the following distribution of times: Given an array in stationarity, when does the last spacer from the ancestral array gets lost? The *expected lineage loss time* is the expectation of this distribution. Analogously, we define the *pairwise loss time distribution* as the distribution of times when two independently evolving lineages lost their last common spacer. In detail, we simulate two lineages starting from a common ancestor and track changes in both lineages simultaneously. *t* is the time when the deletion in one lineage results in the loss of all spacers that are present at that time in the other lineage. The pairwise loss time simulated is then 2*t* since there were two lineages. The distribution is always approximated using 10,000 simulated pairs.

**Figure 5:**
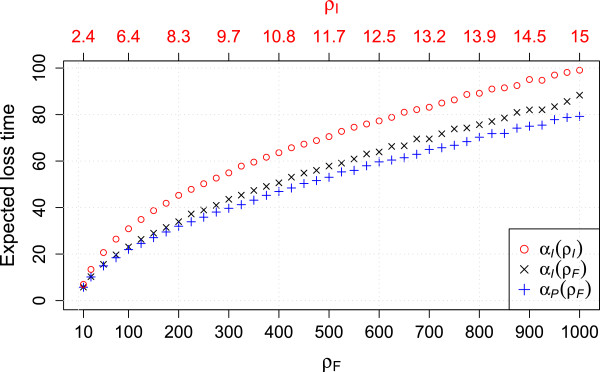
**Expected loss times for both models. ***α*_*l*_ - expected lineage loss time, *α*_*p*_ - expected pairwise loss time. *α*_*l*_(*ρ*_*I*_) = *α*_*p*_(*ρ*_*I*_), thus only one is displayed. Corresponding *ρ*s are in one column, i.e., they result in the same expected length. Each point represents 10,000 simulations.

The expectations of these distributions is denoted by *α*_*l*_(*ρ*_*I*_) (expected lineage loss time under the independent loss model given *ρ*_*I*_), *α*_*p*_(*ρ*_*I*_) (expected pairwise loss time for the independent loss model given *ρ*_*I*_), and analogously with subscript *F* for the fragment loss model. In case the underlying *ρ* is clear, the argument is omitted. The expected lineage and pairwise loss times are lower for the fragment loss model (Figure 5). In the estimation, we set $\hat{\tau }=\alpha_{p}(\hat{\rho })$ for a pair without overlap. Note that this is an underestimate of the time between two arrays since the loss time is an estimate of the minimum, i.e., the first time when two arrays lost common spacers.

### Simulation

Simulation under each model is implemented in a python program. Input is a phylogeny with branch lengths, *ρ* and the type of the model. An ancestor length is drawn at the root of the phylogeny from the stationary distribution of the length model. Spacers are labelled arbitrarily. Then the tree is traversed in preorder and the descendent of each branch given its ancestor and branch length *t* is simulated as follows.

Start with the ancestor *s*, *n* = |*s*|, current time *t*_*c*_ = 0. 

1. Determine the time until the next event of each type: 

(a) Draw a waiting time until the next insertion event from an exponential distribution with rate *λ*.

(b) Draw the waiting times until the next deletion for each spacer or fragment.

(b1) If the independent loss model is simulated, draw *n* exponential waiting times, each with rate *μ*.

(b2) If the fragment loss model is simulated, draw $\frac{n(n+1)}{2}$ exponential waiting times with rate *μ*, one for each fragment.

2. Find the minimal time *t*_min_ over all times generated in step 1.

3. *t*_*c*_ = *t*_*c*_ + *t*_min_.

4. If *t*_*c*_ > *t*, return *s* as the sequence at the descendent node.

5. Else the event that corresponds to *t*_min_ is realized, the other events are discarded. If *t*_min_ corresponds to an insertion, one spacer with a new name is inserted. In case of the unordered model, the spacer is inserted at a random position, in the other cases it is always inserted in the beginning of *s*. If *t*_min_ corresponds to a deletion, modify *s* by deleting the corresponding fragment or spacer.

6. Continue at step 1 with the modified *s*.

### Phylogeny computation using CRISPR distances

The sum of the estimated times given two strains, *τ*, can be interpreted as the distance between these two strains. These distances can be used to compute a distance-based phylogeny using neighbor joining [32] as was presented by Huson and Steel [33]. For the non-reversible models, however, there is more information available, since there is an estimate for the distance of the last common ancestor to each of the two strains. We use a modified neighbor joining method to utilize this information and refer to it as *rooted neighbor joining*. We describe the algorithm with an example.

**Input:** For *k* taxa, all k2 pairs with rooted time estimates, that is *d*_*x*, *y*_ for the distance to taxon *x* from the ancestor of the pair (*x*, *y*).

**Output:** A rooted phylogenetic tree with times *t*.

### Algorithm

1. Compute the weights for all pairs (*x*,*y*):

$$\begin{array}{c} w_{x,y}=\underset{z\neq x,y}{\sum }(d_{x,z}-d_{x,y}+d_{y,z}-d_{y,x})\\ \;=(2-k)(d_{x,y}+d_{y,x})+\underset{z\neq x,y}{\sum }(d_{x,z}+d_{y,z}) \end{array}$$

2. Choose the pair with maximal weight *w*_*x*,*y*_. Create a new node *r* that is the ancestor of (*x*, *y*) with *t*_*r*,*x*_ = *d*_*x*,*y*_ and *t*_*r*,*y*_ = *d*_*y*,*x*_.

3. Compute the distances between all other nodes *z* and *r*:

$$d_{r,z}=\frac{1}{2}(d_{x,z}-d_{x,y}+d_{y,z}-d_{y,x})\text{,}\;d_{z,r}=\frac{1}{2}(d_{z,x}+d_{z,y})$$

4. If only one node is left, return it as the root, else continue with step 1.

By construction, the method results in the correct rooted tree if the distances were extracted from a rooted tree. We show this for three taxa.

For three taxa, there is only one clade, we choose (1,2) to be the correct clade. Then the branch lengths are given in Figure 

Iteration 1: 

Weights: *w*_1,2_ = -(*d*_1,2_ + *d*_2,1_) + *d*_1,3_ + *d*_2,3_ = -(*a* + *b*) + *a* + *c* + *b* + *c* = 2*c*, *w*_1,3_ = -(*a* + *c* + *d*) + *a* + *d* = -*c*, *w*_2,3_ = -(*b* + *c* + *d*) + *b* + *d* = -*c*. Thus for all possible *a*, *b*, *c*, *d*, *w*_1,2_ = argmax_*i*,*j*_*w*_*i*,*j*_ and the correct grouping is chosen by the algorithm.

Create node 4 with *t*_4,1_ = *d*_1,2_ = *a* and *t*_4,2_ = *d*_2,1_ = *b*. The tree is now (t1:a,t2:b)4.

**Figure 6:**
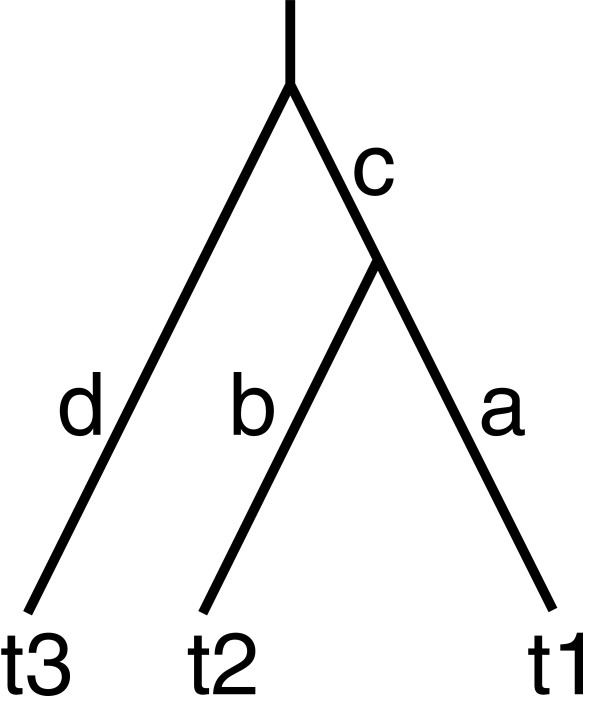
Rooted tree of three taxa with branch lengths.

Iteration 2:

There is only one pair, create node 5 with *t*_3,5_ = *d*_3,4_ = *d* and *t*_4,5_ = *d*_4,3_ = *c*. The resulting tree is ((t1:a,t2:b)4:c,t3:d)5. Apart from the internal labels, this tree is identical to the original one (Figure 6).

We abbreviate the method rooted neighbor joining with times from the fragment loss model by *RNJ*_*F*_, analogously for *NJ* and subscript *O* for the ordered model and subscript *U* for the unordered model.

### *Yersinia pestis* data set

We downloaded available *Yersinia pestis* genomes (final list in Table 2). Unfinished strains were included if open reading frames have been annotated. Cas genes are detected using HMMER [34] and the profiles defined previously for the Ypest type (http://www.ftp.ncbi.nih.gov/pub/wolf/_suppl/CRISPRclass/crisprPro.html[5]). Unfinished strains were excluded if cas genes were detected on different contigs. In these cases, not all cas genes were available.

**Table 2:** **CRISPR arrays from *Yersinia pestis *genomes**

**Strain**	**Accession**	**Yp1**	**Yp2**	**Yp3**
91001	GenBank:NC_005810.1	2^1^-1-0	3-2-1-0	0
a1122	GenBank:NC_017168.1	7-6-2-5-1-4-3-0	4-3-2-1-0	2-1-0
angola	GenBank:NC_010159.1	8-1-4-0		
antiqua	GenBank:NC_008150.1	10-9-1-4-3-0	5-2-0	2-1-0
ca88-4125	GenBank:ABCD00000000.1	7-6-2-5-1-4-3-0	4-3-2-1-0	2-1-0
co92	GenBank:NC_003143.1	7-6-2-5-1-4-3-0	4-3-2-1-0	2-1-0
d106004	GenBank:NC_017154.1	6-2-5-1-4-3-0	3-2-1-0	2-1-0
d182038	GenBank:NC_017160.1	11-6-2-5-4-3-0	3-2-1-0	2-1-0
e1979001	GenBank:AAYV00000000.1	11-6-2-5-4-3-0	3-2-1-0	2-1-0
f1991016	GenBank:ABAT00000000.1	7-6-2-5-1-4-3-0	4-3-2-1-0	2-1-0
harbin35	GenBank:NC_017265.1	4- 3^1^- 0^1^	3-2-1-0	2-1-0
india195	GenBank:ACNR00000000.1	7-6-2-5	4-3-2-1-0	2-1-0
kim10	GenBank:NC_004088.1	4-3-0	3^1^-2-1-0	2-1-0
mg05-1020	GenBank:AAYS00000000.1	7-6-2-5-1-4-3-0	4-3-2-1-0	2-1-0
nepal516	GenBank:NC_008149.1	0^2^	3-2-1-0	2-1-0
pestoidesa	GenBank:ACNT00000000.1	2-1- 0^3^	6-3-2-0	1- 0^1^
pestoidesf	GenBank:NC_009381.1	12-5-1-4-3-0	9-8-6-1-7-0	4-3-2-1-0
pexu2	GenBank:ACNS00000000.1	7-6-2-5-1-4-3-0	4-3-2-1-0	2-1-0
z176003	GenBank:NC_014029.1	6-2-5-1-4-3-0	3-2-1-0	2-1-0

Spacers are grouped and assigned a unique number for each array if they show >90*%* sequence similarity. Different variants are marked by superscript and ignored in the analysis. The leader-proximal end is displayed on the left. Spacer sequences can be found in Additional file 2 for Yp1, in Additional file 3 for Yp2 and in Additional file 4 for Yp3.

 The whole locus was extracted, i.e., the sequence from the start of *cas1* until the end of *csy4*. Nucleotide sequences from the resulting 19 strains were aligned using clustalw [35] into an alignment of 8555 sites that is subsequently used for phylogeny estimation with iqpnni [36].

Putative CRISPR arrays for the 19 strains are extracted using CRISPRfinder [37]. True CRISPR elements are found by comparing the repeat sequence to the known *Yersinia pestis* repeat. The three types of CRISPR arrays are distinguished by their last degenerated repeat [8]. In total, four CRISPR arrays are missing from the CRISPRfinder results. In these cases, we located the respective leader in the genome and extracted repeats and spacers manually. These arrays harbor none or one spacer. For each data set, spacers were assigned the same identity if they show more than 90% sequence similarity. This is a natural cutoff to choose since there was no pair of spacers with similarity between 65% and 90%. Spacer sequences can be found in Additional file 2 for Yp1, in Additional file 3 for Yp2 and in Additional file 4 for Yp3.

## Results

### Parameter estimation for simulated pairs

In the first simulation setting, we present basic simulations with clocklike two-taxon trees. A tree height of 1, 5 and 10 is investigated, resulting in *τ* = 2, 10, 20, and different possible values for *ρ*: *ρ* = 10, 50, 100 for the fragment loss model and *ρ* = 2.4, 4.8, 6.4 for the independent loss model. These values were chosen because they are corresponding *ρ*s (Figure 3).

**Figure 7:**
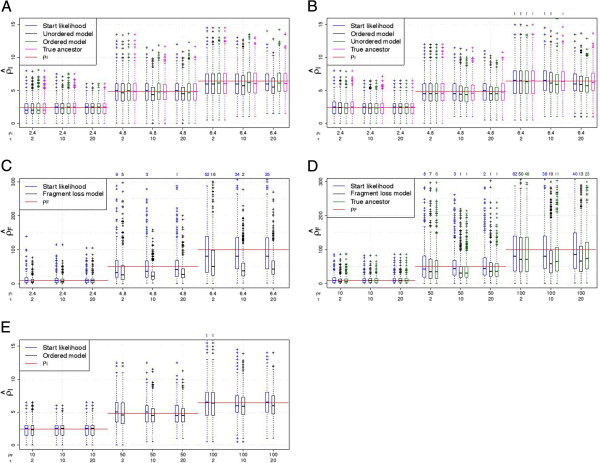
**Estimation of *ρ *with 2 arrays.** (**A**) Simulations under the unordered model. (**B**, **C**) Simulations under the ordered model. (**D**, **E**) Simulations under the fragment loss model. A standard boxplot is shown. 1000 replicates are simulated under each setting. If present, the number of points outside the plot are listed above.

First, we compare the simulated *ρ* with its estimation. *ρ* is estimated based on the start likelihood using the stationary distribution or on the full likelihood summing over all pairs. Note that the start likelihood functions are equal for both independent loss models. The estimates based on the start likelihood and on the full likelihood are very similar for the independent loss models (Figure 7A, B). For the fragment loss model, *ρ*_*F*_ tends to be underestimated for the full likelihood but not for the start likelihood (Figure 7D). The segmentation of ancestors and descendants into independent pairs may cause this bias. This segmentation ignores the probability of deletion events spanning multiple segments and can result in an overestimation of *μ*_*F*_ and thus in an underestimation of the ratio *ρ*_*F*_ = *λ* / *μ*_*F*_.

We also compare the estimation using the full likelihood with the ancestor fixed to the true ancestor and using the full likelihood with summing over possible ancestors. The estimated values of *ρ* are very similar, which leads to the conclusion that the ancestor enumeration works appropriately.

Next, we use the same simulated data sets, but investigate the results when using an incorrect model for the estimation. We only compare the models with single deletions among each other and the models with polarized insertions among each other (see also Table 1). The independent loss models differ only in their insertions. When using the incorrect insertion model, ${\hat{\rho }}_{I}$ is very similar (Figure 7A, B). These models are also very similar in their construction. They are the same if after the first shared spacer there are no spacers unique to one strain. When using the incorrect deletion model, the corresponding *ρ* tends to be estimated (Figure 7C, E). In detail, the *ρ*_*I*_ that is estimated under the ordered model from the data generated under the fragment loss model is on average the *ρ*_*I*_ corresponding to *ρ*_*F*_ used for the simulations (red line in Figure 7E). The underestimation of *ρ*_*F*_ is even present to a larger extent when *ρ*_*F*_ is estimated from data generated under the ordered model compared to the estimation under the true model.

**Figure 8:**
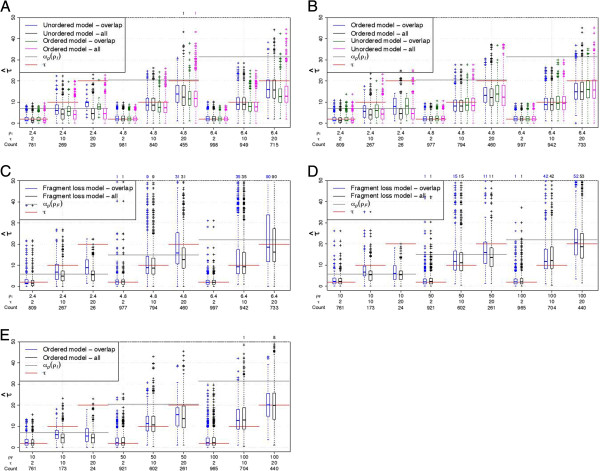
**Estimation of times with 2 arrays.** (**A**) Simulations under the unordered model. (**B**, **C**) Simulations under the ordered model. (**D**, **E**) Simulations under the fragment loss model. *τ* is the sum of the times from the ancestor to both descendants. Only pairs with overlap are included for “overlap”, the number of pairs is given by “Count”. A standard boxplot is shown. 1000 replicates are simulated under each setting. If present, the number of points outside the plot are listed above.

Times can only be estimated for pairs with overlap. The quality of the time’s estimation depends on the simulated *ρ* since the loss times depend on *ρ*. For larger *ρ*, the pairwise loss time is larger, thus it is possible to estimate larger times. When only pairs with overlap are considered, the times tend to be underestimated when the true time exceeds the expected pairwise loss time (Figure 8, blue and green boxes). We use the expected pairwise loss time as an approximation of the times for the empty pairs. Thus for these pairs, $\hat{\tau }=\alpha_{p}(\hat{\rho })$. Using $\hat{\tau }$ from all pairs instead

from the pairs with overlap only, decreases the average time estimated, if there are many empty pairs (Figure 8). This can be explained by two effects. First, the loss time is a minimum, i.e., the first time when two arrays lost common spacers. Second, shorter arrays occur more often among the pairs without overlap. That means, $\hat{\rho }$ is smaller for these pairs and thus their loss time is smaller as well (Table 3).

**Table 3:** **Median *ρ *estimates**

***ρ***	***τ***	***n***_***o***_	$\hat{\rho }$	${\hat{\rho }}_{o}$	${\hat{\rho }}_{e}$
**Unordered model**					
2.4	2	781	2.000	2.500	0.500
2.4	10	269	1.861	3.000	1.491
2.4	20	29	1.833	3.552	1.833
4.8	2	981	4.631	4.872	0.788
4.8	10	840	4.352	4.669	2.191
4.8	20	455	3.846	4.965	3.351
6.4	2	998	6.000	6.000	1.000
6.4	10	949	5.716	5.862	2.326
6.4	20	715	5.285	5.928	3.757
**Ordered model**					
2.4	2	809	2.424	2.500	0.500
2.4	10	267	1.861	2.950	1.500
2.4	20	26	1.833	3.282	1.833
4.8	2	977	4.500	4.552	0.500
4.8	10	794	4.346	4.761	2.564
4.8	20	460	3.963	5.128	3.108
6.4	2	997	6.431	6.431	0.500
6.4	10	942	6.144	6.361	2.598
6.4	20	733	5.625	6.286	3.846
**Fragment loss model**					
10	2	761	6.437	9.201	2.039
10	10	173	9.011	9.832	9.011
10	20	24	9.187	8.705	9.187
50	2	921	35.040	39.779	9.011
50	10	602	31.272	33.123	26.442
50	20	261	36.708	34.167	36.708
100	2	965	71.452	72.466	25.464
100	10	704	57.810	62.657	44.085
100	20	440	66.145	66.145	66.560

Estimation with two arrays under the correct model, same data as Figures 7 and 88. *n*_*o*_ - number of pairs with overlap,${\hat{\rho }}_{o}$ - median of*ρ* estimates of pairs with overlap only,${\hat{\rho }}_{e}$ - median of *ρ* estimates of pairs without overlap only.

Using the true model, we find that times are well estimated until a threshold depending on the simulated *ρ*. For example, for the independent loss model, for *ρ* = 4.8, only *τ* = 2 is well estimated, but for *ρ* = 6.4, *τ* = 2 and *τ* = 10 are well estimated. This threshold is below the expected pairwise loss time. Time estimation for the fragment loss model is more noisy and a slight overestimation can occur for intermediate times that may be related to the underestimation of *ρ* for these parameter settings (Figure 8D).

Time estimates for the incorrect independent loss model are very similar (Figure 8A, B). In general, the ordered model results in slightly lower time estimates. Small and intermediate times are overestimated when the ordered model is applied to data generated under the fragment loss model (Figure 8E), possibly because more events are necessary to explain this data. Applying the fragment loss model to ordered independent loss data also results in an overestimation for intermediate times (Figure 8C).

### Parameter estimation for simulated phylogenies

Next we apply the estimation to data sets simulated on a phylogeny. The same values of *ρ* as in the previous simulations were used. Phylogenies of 10 taxa are generated under a Yule process and rescaled to a specific tree height (tree height of 1, 5, 10, 20 and 30, respectively).

**Figure 9:**
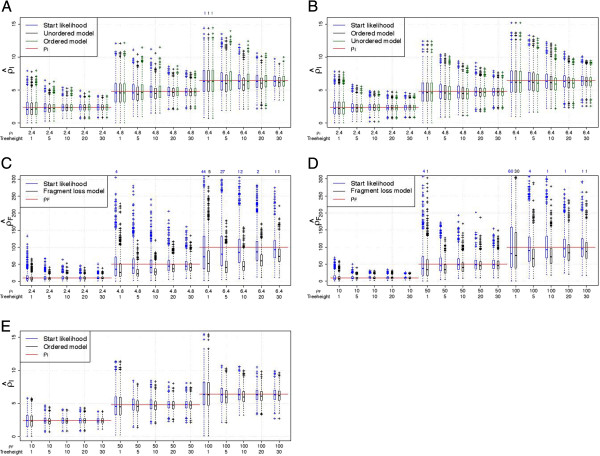
**Estimation of *ρ *with 10 arrays.** (**A**) Simulations under the unordered model. (**B**, **C**) Simulations under the ordered model. (**D**, **E**) Simulations under the fragment loss model. Data was simulated on random Yule trees rescaled to a specific treeheight. A standard boxplot is shown. 1000 replicates are simulated under each setting. If present, the number of points outside the plot are listed above.

**Figure 10:**
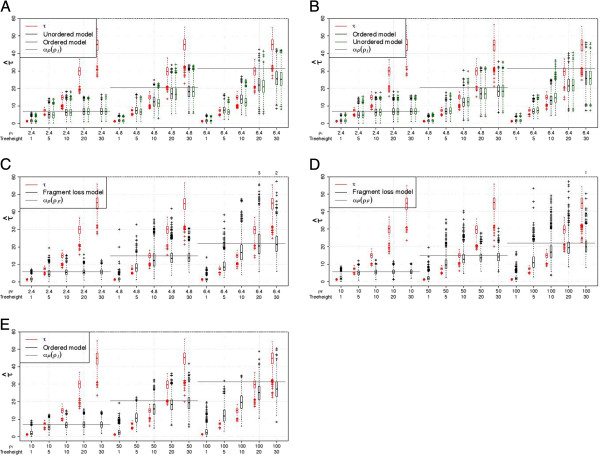
**Estimation of times with 10 arrays.** (**A**) Simulations under the unordered model. (**B**, **C**) Simulations under the ordered model. (**D**, **E**) Simulations under the fragment loss model. A standard boxplot is shown. 1000 replicates are simulated under each setting. If present, the number of points outside the plot are listed above.

These results generally confirm the results for pairs of arrays, but resulting distributions of$\hat{\rho }$ and$\hat{\tau }$ have a lower

 variance. The variance in the estimates is higher for the fragment loss model compared to the independent loss models. For the independent loss model, the mean of the$\hat{\rho }$-values is usually close to*ρ* (Figure 9). Under the fragment loss model, *ρ* for the intermediate times are underestimated (Figure 9D). Times are again well estimated until a threshold depending on the simulated *ρ* (Figure 10). For the fragment loss model, times are overestimated for intermediate tree heights (Figure 10D).

### *Yersinia pestis* analysis

*Yersinia pestis* genomes generally harbor three CRISPR arrays types, called Yp1, Yp2, and Yp3. All three array types have the same repeat sequence and only one set of cas genes of the Ypest type is present in the genome. We demonstrate the methods using three *Yersinia pestis* data sets (Table 4). One data set was assembled from 19 sequenced genomes (see Materials and Methods and Table 2). Pourcel et al. [8] investigate 62 strains but Yp1 is only present in 60 of them. They sequence Yp2 in 15 of them but give no detailed information about Yp3, thus it was not included. Cui et al. [27] investigate 131 strains, including published genomes and *Yersinia pestis* isolates from Asia. The three arrays are present in all of them but sequence information for Yp2 and Yp3 is missing in 6 and 5 strains, respectively.

**Table 4:** ***Yersinia pestis *data sets**

**Data set**	**Array**	**Strains**	**Avg. length**	**Avg. overlap**
	Yp1	19	5.737	0.658
1 (Table 2)	Yp2	19	4.211	0.741
	Yp3	19	2.789	0.865
2 [8]	Yp1	60	6.8	0.905
	Yp2	15	4.733	0.847
	Yp1	131	6.542	0.588
3 [27]	Yp2	125	4.584	0.814
	Yp3	126	2.99	0.931

The average overlap is computed as the mean of the overlap over all pairs. The overlap of a pair is the number of equal spacers divided by the mean length of both arrays.

Data set 1 consists of on average shorter arrays than the published data sets. This results in lower estimates of *ρ* for this data set (Table 5). For data sets 2 and 3, *ρ* estimates between the data sets for the same CRISPR array type are largely congruent. Comparing average times between arrays with different *ρ* is problematic, since larger *ρ* can resolve larger times. Thus we compare average diversity between data sets. Diversity for a pair is computed as the

**Table 5:** ***Yersinia pestis *results**

		**Unordered model**			**Ordered model**			**Fragment loss model**		
**Data**			**Avg.**	**Avg.**		**Avg.**	**Avg.**		**Avg.**	**Avg.**
**set**	**Array**	${\hat{\rho }}_{I}$	**time**	**diversity**	${\hat{\rho }}_{I}$	**time**	**diversity**	${\hat{\rho }}_{F}$	**time**	**diversity**
	Yp1	5.555	5.802	0.2271	5.527	5.947	0.2362	46.24	4.96	0.3331
1	Yp2	4.027	3.479	0.2188	4.005	3.538	0.2241	22.87	3.645	0.3108
	Yp3	2.667	1.506	0.1755	2.667	1.486	0.1772	11.36	1.313	0.2061
2	Yp1	6.625	4.726	0.1445	6.624	4.761	0.1446	90.21	3.859	0.188
	Yp2	4.676	2.984	0.1494	4.655	3.082	0.156	28.61	3.701	0.2748
	Yp1	6.401	9.138	0.2943	6.36	9.259	0.2983	80.76	9.741	0.4408
3	Yp2	4.613	3.329	0.1707	4.607	3.379	0.1749	39.31	3.359	0.2492
	Yp3	2.969	0.7221	0.07339	2.959	0.7184	0.07114	12.6	0.8776	0.1025

 time between the arrays divided by the pairwise loss time, where maximum diversity is 1. Yp3 has the lowest diversity for each data set it is present. However, the results for the other array types differ. Pourcel et al. [8] argued that Yp1 is the most dynamic CRISPR locus. Based on the data of Pourcel et al. (data set 2), diversity is similar in Yp1 and Yp2 under the independent loss model, and diversity is lower in Yp1 than in Yp2 under the fragment loss model. This discrepancy is resolved when comparing the average times. The average time is larger in Yp1 than in Yp2 for each model. Thus there are more events present in Yp1 compared to Yp2. When considering that the longer arrays in Yp1 could resolve larger times, the diversity results in a similar value. Data set 3 [27] shows higher diversity in Yp1 compared to Yp2 under all three models. Diversity in Yp1 is also higher in data set 3 compared to data set 2. Data set 3 thus captures a larger fraction of the diversity in CRISPR spacer content present in *Yersinia pestis*.

**Figure 11:**
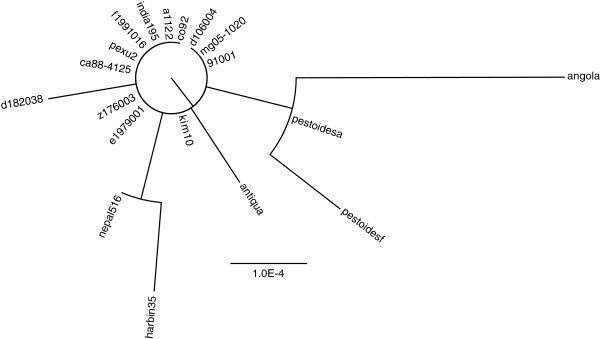
**Phylogeny of the Cas locus from 19 *Yersinia pestis *genomes.** Tree images are created by FigTree [39].

**Figure 12:**
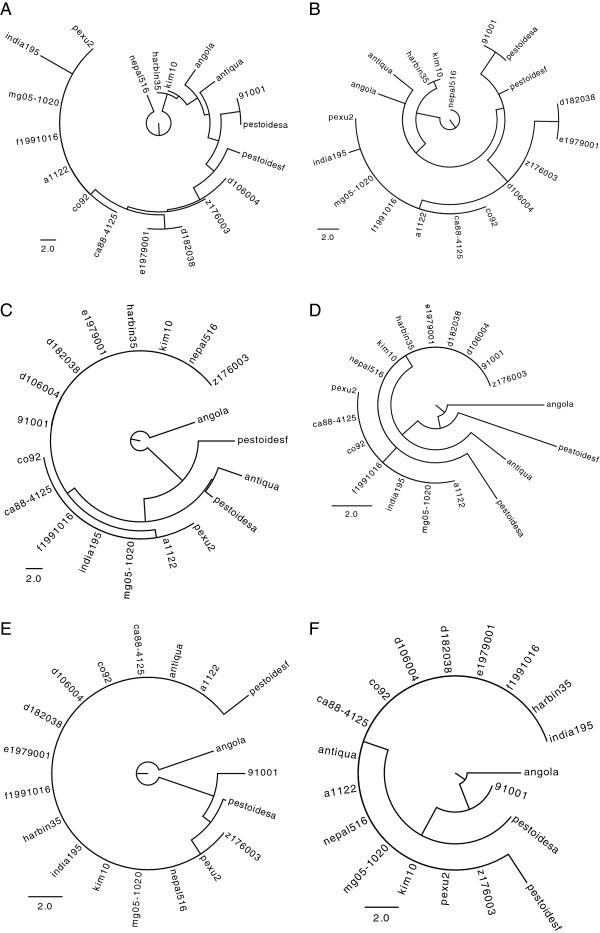
**Trees using the CRISPR spacer data from data set 1. (A,C,E) ***NJ*_*U*_: Neighbor joining tree of times from the unordered model. **(B,D,F)***RNJ*_*F*_: Rooted neighbor joining tree of times from the fragment loss model.**(A,B)** Yp1, **(C,D)** Yp2, **(E,F)** Yp3. Branch lengths correspond to the number of events under the specific model. For clarity, the unrooted neighbor joining trees are shown with the root at the same branch as the rooted neighbor joining tree.

Cas sequence data is only present for data set 1. The respective cas phylogeny contains few substitutions (Figure 11). The spacer distances are also displayed in a tree structure using the unrooted and rooted neighbor joining method (Figure 12). These trees contain substantially more changes than the cas gene phylogeny and there are also few incongruencies. The group (nepal516, harbin35) present in the cas phylogeny is not present in any CRISPR tree, but is compatible with the trees from Yp2 and Yp3. The group (pestoidesa, pestoidesf, angola) is contradicted in all trees. The rooted method tends to connect strains with few spacers directly to the root, for Yp1 this is nepal516 (having only one spacer) and for Yp2 and Yp3 this is angola (having an empty array). Note that the angola strain was indeed described to be a deep-rooting *Yersinia pestis* strain [38]. For the slowly evolving locus Yp3, the clusters displayed by *NJ*_*U*_ and *RNJ*_*F*_ are equal, only the branch lengths differ and the clusters display the relationships well. In detail, there is a cluster for all strains having spacer 0, for all strains having spacer 1, and for all strains having spacer 2. The terminal branch leading to angola is much longer for *NJ*_*U*_, since multiple deletions are needed that can be explained by only one event under the fragment loss model. On the other hand, the branches leading to pestoidesf have about the same length since there are only two observed insertions. For the other more diverse loci, trees display which strains are more divergent and which ones are more similar. For example,*RNJ*_*F*_ for Yp2 shows that angola, pestoidesf, antiqua and pestoidesa are more divergent, whereas the other strains are more similar to each other. Indeed, to convert between two of the other strains at most one event is needed, wheres to convert one of the four strains mentioned into any other one at least two events are necessary.

## Discussion

We present a new method for analyzing CRISPR spacer data from microbial populations. The evolution of CRISPR is mainly driven by the insertion of new spacers during infection with foreign DNA and by the presumably random deletion of successive spacers. We try to meet these biological characteristics in the models presented here. Estimating insertion and deletion rates and time in number of expected events in one lineage allows for comparisons of empirical data sets that could lead to relevant conclusions. First, bacterial groups in different environments can be compared in terms of CRISPR dynamics to assess the relative importance of CRISPR in

 these environments. Different CRISPR array types might show different dynamics and thus have different utility for strain typing. An observed switch in spacer dynamics on a phylogeny might suggest a change in CRISPR cost or environment.

The three models presented here capture different mechanisms of CRISPR evolution, namely polarized addition of spacers and deletion of multiple successive spacers (Table 1). The CRISPR spacer arrays used for the analysis are assumed to be homologous. CRISPR homology can be determined by synteny in genomic positions and by repeat and leader similarities.

Models are necessarily a simplification of the past biological process. In our model, we ignore population dynamics. Our insertion and deletion rates are, as the substitution rates in phylogenetics, a compound parameter including the process of random changes and selection. The model is based on a time-homogenous Markov process and the dynamics are assumed to be in stationarity. Since an analysis is based on one species and one CRISPR type, it is reasonable to assume that the mechanistic insertion and deletion rates are homogeneous across the set of strains analyzed. We can not exclude, however, that subsets of strains experienced a different environment and thus different selection pressure on their spacer content. Simulations showed that the number of spacers in an array is determined mainly by internal parameters, like spacer insertion rate and cost of having spacers, not by external parameters, like the number of viruses in an environment [15].

We are not aware of previous publications estimating parameters under the ordered or the fragment loss model. The length model of the independent loss model corresponds to an *M* / *M* / *∞* queuing model [28]. The unordered model corresponds to the gene content model for the maximum-likelihood distance estimation in [33].

 In the context of birth and death processes it is known as the simple death-and-immigration process (e.g., [40]).

The times estimated under these models also allow a comparison to substitution rates if sequence data is available. This analysis is however complicated by several facts. First, microbial genomes often harbor multiple CRISPR arrays. As a consequence, it is not clear how to combine these estimates to make a comparison possible. Second, spacer content might be different for very closely related strains. Then only a few polymorphisms are available and the substitution rate cannot be estimated reliably. Finally, frequent horizontal gene transfer of the CRISPR/Cas system has been suggested (e.g., [41]), and thus CRISPR rates can only be compared to substitution rates of cas genes.

The parameter estimation as presented here does not use an explicit phylogeny. This is advantageous since no search through tree space is necessary or no precomputed phylogeny needs to be given. The latter may

 not be possible since no external information might resolve the CRISPR relationships. On the other hand, only a distance-based approach is available to display the CRISPR relationships. We can use the rooted distance from non-reversible models to compute rooted non-clocklike distance-based trees.

We find that estimation of the rate parameter performs well on average, but the estimates under the independent loss models show a lower variance. The fragment loss model tends to an underestimation and may be affected by the factorization of the likelihood function. The time estimates are most accurate for shorter times. For longer times, the absence of overlap complicates an accurate time estimation. In the analyses presented here, the different models result in similar estimates. If the incorrect loss model is applied, the corresponding *ρ* tends to be estimated fairly accurately. There is also no clear bias that affects the time estimation under an incorrect model. Note that there is a wide range of possible models accounting for fragment deletions. We chose one with the same instantaneous rate for each possible fragment, i.e., ignoring fragment length. This simplification is mainly for computational reasons. Future work on other fragment loss models, including lengths of fragments, might lead to a better fit for CRISPR spacer data.

We compare the estimations between data sets and between different CRISPR arrays present in a genome. Three *Yersinia pestis* data sets were chosen since they harbor three CRISPR array types and thus this data sets allows for comparison between data sets and between CRISPR array types evolving with different dynamics. Using this data set, we find *ρ* estimates to be similar using two published data sets but lower in a data set assembled from published genomes. Time and diversity estimates differ between data sets thus the presented methods allow comparisons of the diversity of CRISPR loci sampled from different populations.

For the *Yersinia pestis* data from published genomes, we observe only few differences in the cas gene sequences but a high diversity at the spacer level. Thus substitution

 rates cannot be compared with reliability, but nucleotide and CRISPR spacer data provide phylogenetic information at very different time scales. It is possible to compute cas gene phylogenies on the species level (e.g., [41]). In contrast, spacer information could be utilized for closely related strains that have only few differences in the other nucleotide sequences, which has already been done in using CRISPR in strain typing (e.g., [8],[10],[11]). The method presented here can be used to define groups based on the clustering or to find relationships between groups.

A steadily growing literature suggests many other possible mechanisms of CRISPR evolution apart from polarized addition and fragment deletion. Spacer insertion can happen together with an internal deletion [42], or at an internal repeat [43]. Spacers or whole fragments may be duplicated [44]. And present spacers can guide the acquisition of new spacers from the same DNA molecule [45]. Note that these results affect only the insertion step of the CRISPR evolution process. But the fragment deletion model as it is presented here is based on the polarized insertion assumption. Combining an unordered insertion with a fragment deletion process is currently infeasible. Given these studies and the fact that the models presented here do not give substantially different results, the unordered model may be a robust choice for estimating rate and time parameters from CRISPR array data. Note that several simplifications are possible for the likelihood computation under this model. First, for the start likelihood, the estimate of the Poisson parameter is well known to be the mean of the data values. Second, it is reversible, thus only the time between two arrays can be estimated and the ancestor generation can be omitted. Third, the loss time can be calculated analytically and does not need to be acquired using simulations. To make the model comparisons fair, the same computational approach is used for all models in this paper. But it is possible to implement a more efficient approach for the unordered model. Under this model, an algorithm for the likelihood computation on a phylogeny is also potentially feasible.

## Conclusions

We present different models specific for CRISPR spacer content evolution. The three models differ in two aspects. First, fragment loss models differ from the independent loss models since they allow the loss of a succession of spacers in one event. Second, the unordered independent loss model differs from the others since spacers can be incorporated throughout the array, not only on one end. A probabilistic model for each of these three models is presented here. We developed an approach derived from a well behaved stationary distribution, to establish the bounds on the state space that is *a priori* infinite. We find that the simpler model, without fragment deletions, is more robust. Distance-based phylogenies can be calculated from the time estimates, but the rapid change of spacer content restricts this method to closely related strains with similar spacer content.

In summary, the models facilitate quantitative statements about the spacer dynamics of microbial communities. Thus comparisons are possible, for example, between strain collections from one species at different locations or between different homologous CRISPR arrays in the same set of species.

## Competing interests

Both authors declare that they have no competing interests.

## Authors’ contributions

JPB and AK designed the project. AK implemented the methods, carried out simulations and estimations and wrote the manuscript. Both authors discussed the results and the manuscript. Both authors read and approved the final manuscript.

## Supplementary Material

Additional file 1 Proof of equation (3). Click here for file

Additional file 2 ***Yersinia pestis *spacer sequences for data set 1 Yp1 in fasta format.** Click here for file

Additional file 3 ***Yersinia pestis *spacer sequences for data set 1 Yp2 in fasta format.** Click here for file

Additional file 4 ***Yersinia pestis *spacer sequences for data set 1 Yp3 in fasta format.** Click here for file
