# Supplementary material for: Probabilistic models for CRISPR spacer content evolution
Source: BMC Evol Biol. 2013 Feb 26;13:54. doi: 10.1186/1471-2148-13-54 (PMC3704272; doi:10.1186/1471-2148-13-54)
Supplement: Additional file 1 — Proof of equation (3). [file 1471-2148-13-54-S1.pdf]

Additional file 1 - Proof of equation (3)

Show by induction $p(n) = \dfrac{(n+1)(n+2)\mu\lambda^n}{2\prod_{i=0}^{n}(\frac{(i+1)(i+2)}{2}\mu+\lambda)} = \dfrac{(n+1)(n+2)\mu\lambda^n}{2\prod_{i=0}^{n}\lambda(\frac{(i+1)(i+2)}{2\rho}+1)} = \dfrac{(n+1)(n+2)}{2\rho\prod_{i=0}^{n}(\frac{(i+1)(i+2)}{2\rho}+1)}$.

The probabilities must satisfy (1) $\sum_{n=0}^{\infty} p(n) = 1$ and (2) in stationarity, the flow out of any state equals the flow into that state.

<u>$n = 0$:</u> The flow out of state 0 is given by $\lambda p(0)$ and the flow out of state 0 by $\sum_{k=1}^{\infty} \mu p(k)$, thus:

$$\begin{aligned} \lambda p(0) &= \sum_{k=1}^{\infty} \mu p(k) \\ \text{Using (1): } \lambda p(0) &= \mu(1-p(0)) \\ p(0) &= \frac{\mu}{\lambda+\mu}. \end{aligned}$$

<u>n, given the equation for $0, ..., n-1$:</u> The flow out of state n is given by

$$\lambda p(n) + \frac{n(n+1)}{2}\mu p(n)$$

and the flow into state n by

$$\begin{aligned} & \lambda p(n-1) + \sum_{k=n+1}^{\infty} (n+1)\mu p(k) \\ = \ & \lambda p(n-1) + (n+1)\mu(1-\sum_{k=0}^{n} p(k)) \\ = \ & \lambda p(n-1) + (n+1)\mu - (n+1)\mu p(n) - (n+1)\mu \sum_{k=0}^{n-1} p(k). \end{aligned}$$

Equating both and bringing $p(n)$ to one side, yields

$$\begin{aligned} p(n)(\lambda + \frac{(n+1)(n+2)}{2}\mu) &= \lambda p(n-1) + (n+1)\mu - (n+1)\mu \sum_{k=0}^{n-1} p(k) \\ p(n) &= \frac{\lambda p(n-1) + (n+1)\mu - (n+1)\mu \sum_{k=0}^{n-1} p(k)}{\lambda + \frac{(n+1)(n+2)}{2}\mu} \\ p(n) &= \frac{2^{n-1}\lambda^n\mu^{1-n}(n+1)(n+1)}{(\lambda+\mu)} \frac{\Gamma\left[\frac{5}{2}+\frac{\sqrt{\mu(-8\lambda+\mu)}}{2\mu}\right]}{\Gamma\left[\frac{5}{2}+\frac{\sqrt{\mu(-8\lambda+\mu)}}{2\mu}+n\right]} \frac{\Gamma\left[\frac{5}{2}-\frac{\sqrt{\mu(-8\lambda+\mu)}}{2\mu}\right]}{\Gamma\left[\frac{5}{2}-\frac{\sqrt{\mu(-8\lambda+\mu)}}{2\mu}+n\right]} \\ p(n) &= \frac{(n+1)(n+2)\mu\lambda^n}{2\prod_{i=0}^{n}(\frac{(i+1)(i+2)}{2}\mu+\lambda)}, \end{aligned}$$

where Γ is the Gamma function $\Gamma(z) = \int\limits_0^\infty t^z e^{-t} \frac{dt}{t} = \frac{1}{z} \prod\limits_{k=1}^\infty \frac{(1+\frac{1}{k})^z}{1+\frac{z}{k}}$.
The last two steps were derived in Mathematica. □
